# Ergonomic Risk Factors for Musculoskeletal Disorders among Ethnic Lychee–Longan Harvesting Workers in Northern Thailand

**DOI:** 10.3390/healthcare10122446

**Published:** 2022-12-04

**Authors:** Parichat Ong-Artborirak, Supakan Kantow, Katekaew Seangpraw, Prakasit Tonchoy, Nisarat Auttama, Monchanok Choowanthanapakorn, Sorawit Boonyathee

**Affiliations:** 1 Faculty of Public Health, Chiang Mai University, Chiang Mai 50200, Thailand; 2 School of Public Health, University of Phayao, Phayao 56000, Thailand; 3 School of Medicine, University of Phayao, Phayao 56000, Thailand

**Keywords:** musculoskeletal disorders, farmer, ethnic, ergonomics

## Abstract

Musculoskeletal disorders (MSDs) are one of the leading causes of occupational injuries and disabilities. The purpose of this study was to assess the prevalence of MSDs and occupational factors affecting MSDs among ethnic lychee–longan harvesting workers in northern Thailand. A cross-sectional study was conducted in the areas of three upper northern provinces of Thailand. The study areas are located in the highlands and rural plains, where many ethnic minority groups live, including Indigenous, Mien, Karen, and Lua. The majority of them work in a farm of perennial fruit trees, mainly lychee and longan. During the harvest season, 404 participants were recruited for the study using the convenience sampling technique. Data were collected using questionnaires that included general information, an ergonomic risk assessment, and a standardized Nordic questionnaire for assessing MSDs in 10 body parts. The average age of lychee–longan harvesting workers was 48.8 years. Almost all (99.5%) reported MSDs in one or more body regions in the previous seven days of work. The prevalence of MSDs was highest in the hands (82.9%), followed by the shoulders (82.2%) and the neck (79.7%). The total ergonomic risk scores, which included awkward posture, heavy carrying and lifting, repetitive activity, land slope, and equipment, were found to be significantly associated with MSDs in part of the neck (AOR = 1.17, 95%CI = 1.11–1.23), shoulder (AOR = 1.15, 95%CI = 1.10–1.21), elbow (AOR = 1.18, 95%CI = 1.12–1.24), hand (AOR = 1.12, 95%CI = 1.07–1.18), finger (AOR = 1.33, 95%CI = 1.24–1.44), upper back (AOR = 1.14, 95%CI = 1.09–1.20), lower back (AOR = 1.16, 95%CI = 1.11–1.22), hip (AOR = 1.11, 95%CI = 1.06–1.15), knee (AOR = 1.18, 95%CI = 1.12–1.24), and feet (AOR = 1.21, 95%CI = 1.15–1.28) when adjusting for ethnicity, sex, age, BMI, and work experience. Many parts of ethnic workers’ bodies have been affected by occupational injuries, with a high risk of upper extremity injury. As a result, using an ergonomic approach to improving the working environment and appropriate posture movement is very beneficial in preventing MSDs among ethnic harvesting workers.

## 1. Introduction

Musculoskeletal disorders (MSDs) have emerged as a major public health issue, establishing a large burden on medical costs and healthcare. MSDs account for 40% of global compensation costs for occupational and work-related injuries and diseases [1]. Globally, in 2016, back and neck pain was the second most common health outcome for work-related disability-adjusted life years (12.27 million; 13.7%), rising 20.1% from 2000 [2]. These health issues affect the ability and productivity of occupational workers, which leads to absenteeism [3]. Physical impairment and dismemberment among people of working age can have a negative impact on their lives and place a significant financial and caregiving burden on their families [4,5].

Work-related MSDs (WMSDs) are a major issue for many workers [4]. One of the factors contributing to an increase in MSDs is poor ergonomic conditions in workstations. The nature of working conditions which require excessive physical exertion and repetitive physical actions such as lifting, catching, or jerking heavy objects may impact muscles and tendons; as a result, the injured physical body parts exert a force on the skeletal muscles, causing permanent pain and muscle degradation [3]. Reducing MSDs among agricultural workers, particularly fresh fruits and vegetables, which are still harvested using labor-intensive approaches, should be a priority [6].

Agriculture and horticulture are the most common occupational groups in Thailand [7]. Most of the horticultural crops grown in Thailand’s northern border areas are perennial fruits that are economic crops such as lychees, longan, and tangerines. Northern Thailand is geographically characterized as a mountainous area, with many hillsides, steeps, and highlands that differ from other regions, and the populations in the areas are culturally diverse, with ethnic groups such as Karen, Lua, Mien, Akha, Hmong, and Thai Yai [8]. Most ethnic people rely on agriculture for a living, such as farming, cultivating horticultural crops in the highlands and hillsides, and working as general labor. According to Thailand’s report on occupational diseases in 2021, the incidence rate of musculoskeletal disease was 175 per 100,000 people, and most informal workers have health problems as a result of excessive physical exertion and workplace injuries [7]. A previous study focusing on MSDs among lychee fruit farmers revealed an 81.3% prevalence [9]. However, occupational factors and MSDs among workers from different ethnic groups have received little attention. Therefore, the purpose of this research was to investigate the prevalence of MSDs and the relationship between ergonomic risk factors and MSDs among ethnic lychee–longan harvesting workers in northern Thailand. The findings of this study can be used to prevent or reduce the severity of MSDs, potentially affecting the daily lives of ethnic workers in Thailand.

## 2. Materials and Methods

### 2.1. Setting and Design

This study is part of the Unit of Excellence Project “Health Promotion and Quality of Life”. Three provinces were selected: Pong District (out of 9 districts), Phayao Province, Bo Kluea District (out of 15 districts), Nan Province, and Mae Tha District (out of 8 districts), Lamphun Province, where lychee and longan were planted and more than half of the ethnics lived. To select one sub-district from each district, a simple random sampling technique by lottery was employed; then, convenience sampling was used to recruit participants for the study through publicity from local agricultural academics and the assistance of community leaders. Inclusion criteria included (1) being a female or male ethnic aged 20 or over; (2) living in the area for at least one year; (3) working in lychee or/and longan orchards with at least one year of experience; (4) not having any pathology of non-work-related skeletal and muscle diseases diagnosed by a physician; and (5) willing to participate in the study. This cross-sectional study was carried out during the harvest season, from March to June 2022.

The sample size was calculated using the Cochran formula [10] with an unknown number, [Z^2^pq/e^2^], a 95% confidence interval (Z = 1.96), a 50% MSD proportion (*p* = 0.5, q = 0.5), and a 5% measurement error (e = 0.05). A 5% increase in the number of samples was added to the study sample to avoid incomplete data. According to the calculation, a total of 404 samples were obtained from a population of three provinces. Because the majority of the sampled participants did not speak Thai, it was necessary to recruit research assistants in each area to conduct face-to-face interviews. These were all community village health volunteers who spoke the local language of each ethnic group. The meeting was scheduled for three hours in order for the researcher to clarify the objectives of the research, the clarification of the research questionnaires, the data collection process, interview scheduling, and the rights and privacy of the study samples, with the goal of ensuring that everyone understood the research process in the same direction.

### 2.2. Instruments

The data were collected using questionnaires that were divided into five parts. Part 1: General information included ethnicity, gender, age, marital status, education, body mass index (BMI), underlying disease, work hour per day, work experience, lifting weight, and frequency of lifting activity per day. Part 2: MSDs during the previous 7 days of work, modified from the Standardized Nordic Questionnaire [11], which consists of 10 body parts: neck, shoulder, elbow, hand, finger, upper back, lower back, hip, knee, and feet. The questions could be answered yes or no, with yes indicating pain and no indicating no pain. Part 3: Questionnaire for ergonomic risk assessment that was developed after reviewing the literature and related research [9,12,13,14]. It contained 26 items divided into 5 domains, Domain 1: Awkward posture—8 items (e.g., you bend over or turn to the left or right while working), Domain 2: Heavy carrying and lifting—5 items (e.g., you lift a fruit basket or carry heavy loads), Domain 3: Repetitive activity—4 items (e.g., you make repetitive movements of your hands, wrists, and arms while working), Domain 4: Land slope—4 items (for example, you work in the areas with land slope or hillsides), and Domain 5: Equipment—5 items (e.g., you use equipment such as bamboo ladders or steel ladders for climbing trees). This was assessed using 3 rating scales: rarely practice = 0 point, sometimes practice = 1 point, and regularly practice = 2 points. The possible scores ranged from 0 to 52 points. A higher ergonomic risk score indicated an increased risk.

The ergonomic risk assessment questionnaire was validated by three experts in ergonomics, public health, and occupational health and safety. The questionnaire was then pilot tested on 30 samples with similar characteristics to the samples in the study. For the reliability test, Cronbach’s alpha coefficient was found to be 0.804. The interview lasted approximately 30 min for each person. The data were checked for accuracy and completeness by the researcher.

### 2.3. Statistical Analysis

The SPSS (SPSS Inc., Chicago, IL, USA) software for Windows was used to analyze descriptive statistics such as frequency, percentage, mean, standard deviation (SD), median, interquartile range (IQR), minimum (Min.), and maximum (Max.). To test the differences in ergonomic risk scores among ethnic worker groups, the one-way ANOVA was used. The proportion of MSDs among ethnic worker groups was compared using the Chi-square statistic. The relationship between ergonomic risk scores classified by domain was examined using Spearman’s rank correlation coefficients (r_s_). The ergonomic risk factors affecting MSDs in body parts were investigated using binary logistic regression with both unadjusted and adjusted models. The adjusted model included the important variables associated with MSDs, including ethnicity, gender, age, BMI, and work experience [15,16,17,18,19]. Odds ratios (ORs) with 95% confidence intervals (95%CI) were presented. The significance level for statistical tests was set at 0.05. 

## 3. Results

The general information about the participants is shown in Table 1. The ethnic groups of lychee–longan harvesting workers were Mian (36.5%), Karen (34.3%), Lua (19.5%), and indigenous (10.8%). The majority of workers were female (51.0%) and married (73.8%). Their mean age and BMI were 48.8 years and 24.5 kg/m^2^, respectively. Most had a primary level of education or less (66.6%) and did not have an underlying disease (51.0%). The average number of years worked in their current job was 9.4, with 6.3 working hours per day. The average lifting weight of a fruit basket was 11.6 kg, and the median frequency of lifting activity was 6 times per day.

Ergonomic risk scores among ethnic worker groups are presented in Table 2. The finding showed a difference in total ergonomic risk score among them (*p* = 0.032). When classified by domain, only the awkward posture factor showed a difference in score (*p* = 0.005).

**Figure 1:**
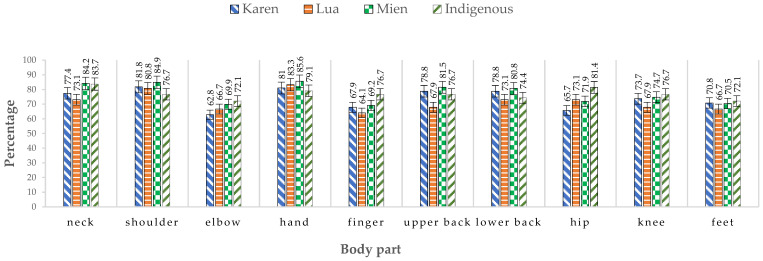
Prevalence of MSDs in body parts for different ethnic worker groups.

Table 3 and Figure 1 show the prevalence of MSDs in 10 body parts over the previous 7 days of work for different ethnic worker groups. Almost all ethnic harvesting workers (99.5%) reported MSDs in one or more body regions in the previous 7 days of work. The prevalence of MSDs was highest in the hands (82.9%), followed by the shoulders (82.2%) and the neck (79.7%). MSDs in 10 body regions showed no difference among ethnic worker groups.

The relationships between ergonomic risk factors classified by domain among ethnic workers are presented in Table 4. There were positive correlations between awkward posture, heavy carrying and lifting, repetitive activity, land slope, and equipment (*p* < 0.01), with Spearman’s rank coefficients (r_s_) ranging from 0.304 to 0.528.

The ergonomic risk factors influencing MSDs in 10 body regions among ethnic harvesting workers are shown in Table 5. In the unadjusted model, total ergonomic risk scores were found to be significantly associated with MSDs in part of the neck (COR = 1.17, 95%CI = 1.13–1.22), shoulder (COR = 1.16, 95%CI = 1.12–1.20), elbow (COR = 1.18, 95%CI = 1.13–1.23), hand (COR = 1.13, 95%CI = 1.09–1.17), finger (COR = 1.34, 95%CI = 1.25–1.43), upper back (COR = 1.17, 95%CI = 1.12–1.21), lower back (COR = 1.16, 95%CI = 1.12–1.21), hip (COR = 1.11, 95%CI = 1.07–1.14), knee (COR = 1.20, 95%CI = 1.15–1.25), and feet (COR = 1.24, 95%CI = 1.18–1.30). Each domain, including awkward posture, heavy carrying and lifting, repetitive activity, land slope, and equipment, was found to be a predictor of MSDs in all 10 body parts (*p* < 0.05). In the model adjusted for ethnicity, sex, age, BMI, and work experience, total ergonomic risk scores were also found to be significantly associated with MSDs in part of the neck (AOR = 1.17, 95%CI = 1.11–1.23), shoulder (AOR = 1.15, 95%CI = 1.10–1.21), elbow (AOR = 1.18, 95%CI = 1.12–1.24), hand (AOR = 1.12, 95%CI = 1.07–1.18), finger (AOR = 1.33, 95%CI = 1.24–1.44), upper back (AOR = 1.14, 95%CI = 1.09–1.20), lower back (AOR = 1.16, 95%CI = 1.11–1.22), hip (AOR = 1.11, 95%CI = 1.06–1.15), knee (AOR = 1.18, 95%CI = 1.12–1.24), and feet (AOR = 1.21, 95%CI = 1.15–1.28). Similarly, all domains were found to be a significant factor of MSDs in all 10 body parts, with the exception of repetitive activity, which was marginally associated with hip (AOR = 1.15, 95%CI = 0.94–1.39).

## 4. Discussion

Almost all ethnic lychee–longan harvesting workers (Karen, Lua, Mian, and Indigenous) in Thailand’s upper northern border region reported MSDs in one or more body regions in the past 7 days during the harvest season, with the most common being physical pains in the hands (82.9%), shoulders (82.2%), neck (79.7%), and lower back (78.0%). The nature of the work requires workers to reach up, climb up trees, bend, and tilt to collect fruits; these activities require excessive physical movements and exertion, resulting in physical symptoms such as strain, pain, stiffness, and swelling on the muscles and in the upper extremity. Due to the fact that workers manually twisted the bunches of lychee–longan from the branches, they were at a higher risk of hand/wrist, shoulder, and neck MSDs. Loading and lifting may also cause back pain. Similarly, a previous study conducted in Thailand found a 7-day prevalence of MSDs among lychee fruit farmers at 81.3% [9]. These can be compared to other studies conducted in workers whose jobs are similar in nature. The occurrence of 7-day WMSDs among Thai coffee farmers was 79.4%, with pains in the neck, shoulder, and wrist being the most common [20]. Pistachio farm workers in southeastern Iran had the highest prevalence of MSDs in the shoulders (63.7%), followed by the lower back (63.0%) and wrists/hands (52.1%) in the past week [21]. The prevalence of 7-day MSDs among oil palm fruit harvesters was found to be 45% in Malaysia [22] and 54.5% in Thailand [23], with lower back disorders being the most reported. The findings indicated that ethnic harvesting workers experience pain and injuries, which may affect their daily physical performance and ability to work, as well as their quality of life.

Our findings revealed ergonomic risk factors in all domains influencing MSDs. This indicated that ergonomic health hazards are an occupational factor that affects ethnic harvesting workers. It was found that a higher score of awkward postures increased the risk of MSDs in all 10 body parts. This can be explained by the fact that their working posture requires use of their upper and lower extremities in order to collect fruits from plantations. The majority of the participants in this investigation used a great deal of head movements in an upward position, arms raised above shoulder level for a long time, and finger and hand/wrist twisted in awkward postures. All of these movements are risk factors for MSDs among fruit harvesting workers. Such awkward movements may result in deformity and long-term injuries [24]. Some studies have found that behavior that uses the upper extremity of the body to work in the overhead position is associated with skeletal muscle problems [25]. A previous study conducted among sugarcane farmers in northeastern Thailand found that working in awkward postures was associated with reporting WMSDs during past 12 months (AOR = 1.95; 95% CI = 1.01–3.77) [26]. In addition, an awkward posture was a particularly significant risk factor of MSDs among oil palm fruit harvesters in Malaysia [22].

The heavy carrying and lifting score was positively associated with the MSDs in all body parts. The workers in lychee and longan plantations lifted and carried heavy baskets vertically repeatedly, with a maximum lifting frequency of 35 times per day and an average lifting weight of 11.6 kg each time, which may reflect inappropriate ergonomic factors. They also transported fruit baskets with a carrying pole. Moreover, physical exertion while working, such as pulling and dragging, was observed in ethnic harvesting workers. According to a prior study, lifting heavy loads was found to be strongly linked to shoulder and neck pain among hill farmers (OR = 3.2, 95% CI = 1.8–5.6) [18]. Lower back MSDs were related to heavy lifting in Thai oil palm harvesting workers (AOR = 3.0, 95%CI = 1.4–6.5) [23]. Some studies have found that heavy lifting has a significant impact on the upper extremity, resulting in back pain and muscle injuries [20,27]. Forceful exertions were also associated with reporting WMSDs in the past year among Thai sugarcane farmers (AOR = 2.8, 95%CI = 1.5–5.0) [26]. Several load-carrying measures, such as duration, frequency, and weight, suggested a link between back pain and disability [28].

A higher score of repetitive movements increased the risk of MSDs, especially in the upper extremity. This can be explained by the fact that the participants in this study used forearm, elbow, wrist, and finger movements excessively and repeatedly to collect fruits, resulting in pain and injury [24]. This is consistent with a previous study that reported that WMSDs in the past year among Thai sugarcane farmers are affected by repetitive motions (AOR = 1.9, 95%CI = 1.1–3.4) [26]. Repetitive movement was associated with shoulder (AOR = 2.0, 95%CI = 1.1–3.5) and neck (AOR = 2.2, 95%CI = 1.1–4.5) MSDs in Thai oil palm harvesters [23]. It also pointed out that performing inappropriate and repetitive postures is a risk factor for developing health problems such as deformity and long-term injuries [14,29,30,31].

In terms of geographical location, fruit tree cultivation occurs in foothill or land slope areas. The results showed that the land slope was one of the factors associated with the occurrence of MSDs in all body parts among workers. Working on slope surfaces, such as lifting fruit baskets to transport to the sorting site, and manual material handling, may result in occupational injuries due to concerns about whole-body stability and slip potential changing their posture and motion profile [32,33]. Ground slope angle can also influence lifting kinematics and kinetics, resulting in different risks [32,33]. This is consistent with previous studies that have found that working on a completely hilly farming terrain was associated with low back pain (AOR = 4.0, 95%CI = 1.2–13.9) among hill farmers in Northern Thailand [18].

The equipment factor can increase the probability of developing MSDs in all body parts. Using scissors in the sorting process to cut the branches and leaves of lychee and longan before packing them into boxes may increase the risk of upper limb injuries such as the finger and hand. Ethnic workers used a 3–5 m bamboo or steel ladder to climb up and down trees to pick lychee and longan, which may cause aches and pains in body parts, especially lower limbs such as the feet and knee. Similarly, carrying a ladder and clipping are ergonomic risk factors in orange harvesters [34]. Among apple harvest workers, activities such as keeping balance on a ladder while picking apples and moving the ladder can contribute to occupational injuries including muscle strains of body parts [13,35]. Besides, the type of ladder, such as steel or bamboo, is a factor that can affect forces in ladder handling due to the different weight of the ladder, and having knowledge of lifting technique may assist a worker in reducing strains [35]. Hand work, particularly cutting or clipping, is an urgent priority in the effort to improve nonfatal injury prevention in agricultural workplaces [34].

There are certain limitations in this study that deserved to be mentioned. Firstly, this study was a cross-sectional study, and the researcher was unable to show causal inferences of the associated factors of MSD-related factors. Secondly, the samples in the study were ethnic minority people recruited by convenience sampling in the community areas of the northern border of Thailand; therefore, the results may not be generalizable to the population. Thirdly, the researcher did not use a scale to measure the severity of the pains and discomfort of the farmers’ physical body. The participants were interviewed and responded according to their experience and symptoms; therefore, there may be a bias in the results of the study. In the future, qualitative research should be considered for the study in order to obtain in-depth information that will assist in the prevention of MSDs among farmers living in rural areas of northern Thailand.

## 5. Conclusions

Our findings revealed that ethnic lychee–longan harvesting workers living in rural northern Thailand commonly experienced pain in all parts of their bodies, notably the upper limbs, such as the hands and shoulders. In addition, MSDs in all body areas were found to be associated with ergonomic risks including awkward posture, heavy carrying and lifting, repetitive movements, land slope, and equipment. These risk factors may contribute to chronic pain and injury, negatively affecting their quality of life. Therefore, policy prevention to support health promotion among ethnic worker groups is important, such as welfare assistance and health promotion programs. Using an ergonomic approach to improving the working environment and appropriate posture and movement are very helpful to prevent MSDs among ethnic workers. A health promotion program related to exercises to build muscle flexibility and strength, as well as occupational safety knowledge to prevent MSDs, should be considered.

## Figures and Tables

**Table 1:** The general characteristics of ethnic lychee–longan harvesting workers.

Participant Characteristics	Total (*n* = 404)	Karen (*n* = 137)	Lua (*n* = 78)	Mien (*n* = 146)	Indigenous (*n* = 43)
Sex					
Male	198 (49.0%)	62 (45.3%)	39 (50.0%)	79 (54.1%)	18 (41.9%)
Female	206 (51.0%)	75 (54.7%)	39 (50.0%)	67 (45.9%)	25 (58.1%)
Age (year)					
Mean (SD)	48.8 (11.7)	52.6 (10.7)	42.4 (11.3)	47.9 (11.3)	50.6 (12.0)
Min–Max	20–73	21–70	22–73	20–71	29–70
Marital status					
Single/Widowed/Separated	106 (26.2%)	28 (20.4%)	21 (26.9%)	50 (34.2%)	7 (16.3%)
Married	298 (73.8%)	109 (79.6%)	57 (73.1%)	96 (65.8%)	36 (83.7%)
Education					
Primary school or lower	269 (66.6%)	99 (72.3%)	46 (59.0%)	94 (64.4%)	30 (69.8%)
Secondary school	135 (33.4%)	38 (27.7%)	32 (41.0%)	52 (35.6%)	13 (30.2%)
BMI (kg/m^2^)					
Mean (SD)	24.5 (3.5)	24.7 (3.3)	24.2 (3.8)	24.4 (3.6)	25.0 (3.2)
Min–Max	15.2–36.2	15.6–33.8	16.0–34.6	17.4–36.2	19.9–32.9
Underlying disease					
Yes	198 (49.0%)	76 (55.5%)	34 (43.6%)	69 (47.3%)	19 (44.2%)
No	206 (51.0%)	61 (44.5%)	44 (56.4%)	77 (52.7%)	24 (55.8%)
Work hour per day (hour)					
Mean (SD)	6.3 (1.5)	6.4 (1.1)	7.0 (1.3)	5.8 (1.6)	6.4 (1.7)
Min–Max	3–10	4–9	4–8	3–9	3–10
Work experience (year)					
Mean (SD)	9.4 (4.8)	9.8 (4.4)	8.0 (5.0)	9.7 (4.8)	9.8 (5.0)
Min–Max	1–25	1–22	1–23	1–25	1–25
Lifting weight (kg)					
Mean (SD)	11.6 (3.4)	12.4 (4.0)	10.0 (0.0)	11.8 (3.7)	10.7 (2.2)
Min–Max	10–25	10–25	10–10	10–25	10–20
Frequency of lifting activity (time)					
Median (IQR)	6 (5)	5 (6)	8 (5)	8 (5)	6 (4)
Min–Max	1–35	1–35	1–25	1–32	1–30

**Table 2:** Comparison of ergonomic risk scores among ethnic worker groups.

Ergonomic Risk	Total (*n* = 404)	Karen (*n* = 137)	Lua (*n* = 78)	Mien (*n* = 146)	Indigenous (*n* = 43)	*p*-Value
Awkward posture (score)						
Mean (SD)	12.2 (2.3)	12.4 (1.9)	11.3 (2.6)	12.4 (2.3)	12.6 (2.3)	0.005 ^†^
Min–Max	5–16	5–16	5–15	5–16	6–16	
Heavy carrying and lifting (score)						
Mean (SD)	7.2 (1.8)	7.4 (1.6)	6.8 (2.1)	7.24 (1.8)	7.18 (1.9)	0.280 ^†^
Min–Max	2–10	2–20	2–10	2–10	2–10	
Repetitive activity (score)						
Mean (SD)	6.3 (1.2)	6.5 (1.2)	6.1 (1.34)	6.31 (1.24)	6.32 (1.28)	0.099
Min–Max	3–8	3–8	3–8	3–8	3–8	
Land slope (score)						
Mean (SD)	6.0 (1.4)	6.1 (1.2)	5.6 (1.7)	6.0 (1.5)	6.1 (1.3)	0.137 ^†^
Min–Max	2–8	2–8	2–8	2–8	3–8	
Equipment (score)						
Mean (SD)	6.5 (1.8)	6.6 (1.6)	6.6 (2.2)	6.4 (1.7)	6.9 (1.7)	0.274 ^†^
Min–Max	2–10	2–10	2–10	2–10	3–10	
Total (score)						
Mean (SD)	38.2 (7.0)	39.0 (6.0)	36.3 (8.4)	38.4 (6.9)	39.2 (6.6)	0.032 ^†^
Min–Max	17–48	17–48	18–48	17–47	17–48	

^†^ Welch’s ANOVA.

**Table 3:** Comparison of MSD prevalence in body regions during the past 7 days of work among ethnic worker groups.

Body Part	Total (*n* = 404)	Karen (*n* = 137)	Lua (*n* = 78)	Mien (*n* = 146)	Indigenous (*n* = 43)	*p*-Value
Neck	322 (79.7%)	106 (77.4%)	57 (73.1%)	123 (84.2%)	36 (83.7%)	0.182
Shoulder	332 (82.2%)	112 (81.8%)	63 (80.8%)	124 (84.9%)	33 (76.7%)	0.627
Elbow	271 (67.1%)	86 (62.8%)	52 (66.7%)	102 (69.9%)	31 (72.1%)	0.540
Hand	335 (82.9%)	111 (81.0%)	65 (83.3%)	125 (85.6%)	34 (79.1%)	0.669
Finger	277 (68.6%)	93 (67.9%)	50 (64.1%)	101 (69.2%)	33 (76.7%)	0.550
Upper back	313 (77.5%)	108 (78.8%)	53 (67.9%)	119 (81.5%)	33 (76.7%)	0.134
Lower back	315 (78.0%)	108 (78.8%)	57 (73.1%)	118 (80.8%)	32 (74.4%)	0.541
Hip	287 (71.0%)	90 (65.7%)	57 (73.1%)	105 (71.9%)	35 (81.4%)	0.225
Knee	296 (73.3%)	101 (73.7%)	53 (67.9%)	109 (74.7%)	33 (76.7%)	0.671
Feet	283 (70.0%)	97 (70.8%)	52 (66.7%)	103 (70.5%)	31 (72.1%)	0.904
All	402 (99.5%)	137 (100.0%)	78 (100.0%)	144 (98.6%)	43 (100.0%)	-

**Table 4:** Spearman’s rank coefficients (r_s_) between ergonomic risk scores classified by domain among ethnic workers.

Ergonomic Risk	1	2	3	4	5	6
1. Awkward posture	1	0.445 *	0.304 *	0.503 *	0.528 *	0.798 *
2. Heavy carrying and lifting		1	0.417 *	0.388 *	0.410 *	0.693 *
3. Repetitive activity			1	0.347 *	0.305 *	0.510 *
4. Land slope				1	0.432 *	0.660 *
5. Equipment					1	0.740 *
6. Total score						1

* Significance at the 0.01 level.

**Table 5:** The ergonomic risk factors associated with MSDs in body parts among ethnic lychee–longan harvesting workers.

Body Part	OR (95%CI)					
	Awkward Posture	Heavy Carrying and Lifting	Repetitive Activity	Land Slope	Equipment	Total
Neck ^†^	1.93 (1.66–2.24)	1.56 (1.36–1.79)	1.73 (1.43–2.10)	1.84 (1.54–2.20)	1.78 (1.52–2.07)	1.17 (1.13–1.22)
Neck ^††^	1.99 (1.65–2.41)	1.37 (1.17–1.60)	1.45 (1.16–1.82)	1.55 (1.27–1.90)	1.58 (1.32–1.90)	1.17 (1.11–1.23)
Shoulder ^†^	1.80 (1.56–2.07)	1.48 (1.29–1.70)	1.83 (1.50–2.24)	1.74 (1.46–2.08)	1.77 (1.51–2.08)	1.16 (1.12–1.20)
Shoulder ^††^	1.85 (1.54–2.21)	1.28 (1.09–1.50)	1.54 (1.22–1.95)	1.48 (1.21–1.82)	1.61 (1.33–1.95)	1.15 (1.10–1.21)
Elbow ^†^	1.85 (1.61–2.12)	1.49 (1.31–1.69)	1.51 (1.27–1.79)	1.75 (1.45–2.01)	1.74 (1.51–2.01)	1.18 (1.13–1.23)
Elbow ^††^	1.97 (1.66–2.32)	1.38 (1.19–1.59)	1.31 (1.07–1.59)	1.50 (1.25–1.80)	1.64 (1.38–1.94)	1.18 (1.12–1.24)
Hand ^†^	1.53 (1.35–1.73)	1.49 (1.30–1.71)	1.60 (1.31–1.94)	1.70 (1.43–2.03)	1.68 (1.44–1.96)	1.13 (1.09–1.17)
Hand ^††^	1.48 (1.26–1.72)	1.33 (1.13–1.56)	1.32 (1.05–1.67)	1.47 (1.20–1.81)	1.53 (1.27–1.85)	1.12 (1.07–1.18)
Finger ^†^	2.31 (1.94–2.75)	1.92 (1.64–2.24)	2.04 (1.68–2.48)	2.42 (1.97–2.98)	2.16 (1.82–2.56)	1.34 (1.25–1.43)
Finger ^††^	2.42 (1.95–3.00)	1.67 (1.41–1.98)	1.71 (1.36–2.13)	2.17 (1.72–2.74)	1.86 (1.55–2.25)	1.33 (1.24–1.44)
Upper back ^†^	1.85 (1.60–2.12)	1.64 (1.43–1.89)	1.60 (1.33–1.93)	1.63 (1.39–1.93)	1.79 (1.54–2.09)	1.17 (1.12–1.21)
Upper back ^††^	1.74 (1.48–2.05)	1.41 (1.21–1.65)	1.26 (1.02–1.56)	1.32 (1.09–1.60)	1.59 (1.33–1.91)	1.14 (1.09–1.20)
Lower back ^†^	1.80 (1.57–2.06)	1.54 (1.35–1.76)	1.49 (1.24–1.79)	1.81 (1.52–2.16)	1.80 (1.54–2.10)	1.16 (1.12–1.21)
Lower back ^††^	1.81 (1.53–2.14)	1.37 (1.18–1.60)	1.23 (1.00–1.53)	1.59 (1.30–1.94)	1.70 (1.41–2.04)	1.16 (1.11–1.22)
Hip ^†^	1.41 (1.27–1.57)	1.36 (1.20–1.53)	1.28 (1.08–1.52)	1.38 (1.19–1.61)	1.60 (1.40–1.83)	1.11 (1.07–1.14)
Hip ^††^	1.40 (1.23–1.60)	1.27 (1.11–1.46)	1.15 (0.94–1.39)	1.25 (1.05–1.49)	1.57 (1.33–1.85)	1.11 (1.06–1.15)
Knee ^†^	1.94 (1.67–2.24)	1.65 (1.44–1.90)	1.59 (1.33–1.91)	1.94 (1.62–2.31)	1.88 (1.61–2.19)	1.20 (1.15–1.25)
Knee ^††^	1.87 (1.57–2.22)	1.44 (1.23–1.68)	1.28 (1.03–1.57)	1.61 (1.32–1.97)	1.65 (1.38–1.97)	1.18 (1.12–1.24)
Feet ^†^	2.14 (1.82–2.51)	1.82 (1.57–2.12)	1.60 (1.34–1.90)	1.98 (1.65–2.36)	1.97 (1.68–2.30)	1.24 (1.18–1.30)
Feet ^††^	2.09 (1.74–2.52)	1.58 (1.34–1.86)	1.24 (1.01–1.52)	1.65 (1.35–2.02)	1.68 (1.41–2.01)	1.21 (1.15–1.28)

^†^ Unadjusted model; ^††^ Adjusted model for ethnicity, sex, age, BMI, and work experience.

## Data Availability

The data presented in this study are available on request from the corresponding author.
